# Differential Roles for Dopamine D1-Like and D2-Like Receptors in Mediating the Reinforcing Effects of Cocaine: Convergent Evidence from Pharmacological and Genetic Studies

**DOI:** 10.4172/2329-6488.1000e124

**Published:** 2015-07-25

**Authors:** Takato Hiranita, Gregory T Collins

**Affiliations:** 1 Division of Neurotoxicology, National Center for Toxicological Research (NCTR), U.S. Food and Drug Administration (FDA), 3900 NCTR Road Jefferson, AR 72079-9501, USA; 2 Department of Pharmacology, University of Texas Health Science Center at San Antonio, 7703 Floyd Curl Dr., Mail Code 7764, San Antonio, TX 78229, USA; 3 South Texas Veterans Health Care System, 7400 Merton Minter Dr, San Antonio, TX 78229, USA

## Abstract

A series of studies by Drs. Barak Caine, James Woods, Gregory Collins, Jonathan Katz and Takato Hiranita demonstrated a novel and unique reinforcing effect using dopamine (DA) D2-like receptor [D2-like R: D_2_, D_3_, and D_4_ receptor subtypes (respectively, D_2_R, D_3_R, and D_4_R)] agonists in rats and genetically modified mice. In order to understand how important their findings are, a comparison was made regarding the reinforcing effects of DA D2-like R full agonists with those of DA uptake inhibitors and of a DA D1-like receptor [D1-like R, D_1_ and D_5_ receptor subtypes (D_1_R and D_5_R)] full agonist (±)-SKF 82958.

## DA transporter (DAT) as a binding site of cocaine

A drug self-administration (SA) procedure has been used to directly assess reinforcing effects of various drugs across pharmacological classes including cocaine under various schedules of reinforcement in various species because of its relative high face and predictive validities [1–3]. Using a drug SA procedure, a number of preclinical and clinical studies have demonstrated substantial implication of DA in reinforcing effects of cocaine [2,4]. Among a large number of other presynaptic and postsynaptic sites that cocaine can bind, an inhibition of DA uptake through the DAT is known to be a primary mechanism underlying reinforcing effects of cocaine [5–8]. In fact, a standard DA uptake inhibitor methylphenidate (see Table 1 for details) maintain SA responding above vehicle levels under a fixed ratio (FR) schedule of reinforcement in naïve rats [9] and when evaluated in rats trained to self-administer cocaine [6,10,11]. Further, pretreatment with standard DA uptake inhibitors also produces a leftward shift of the dose-effect curve (DEC) for cocaine SA, suggesting that they potentiate the reinforcing effects of cocaine [6,10]. [12–14] In addition, cocaine was not reinforcing in mice that underwent a genetic inactivation of the DAT [knockout (KO) and knockin (KI)] [7,8].

## SA of direct DA receptor ligands

### A DA D1-like receptor [D1-like R: D_1_ and D_5_ receptor subtypes (respectively, D_1_R and D_5_R)] direct full agonist

As with the DA uptake inhibitor methylphenidate [15], the DA D1-like R full agonist (±)-SKF 82958 was reliably reinforcing under an FR schedule of reinforcement in drug naïve rats [16] and rhesus monkeys [17]. When substituted for cocaine, there are mixed results. For example, (±)-SKF 82958 maintained SA responding above vehicle levels in mice [18,7] and rhesus monkeys [19] when substituted for cocaine. However, a range of the reliably reinforcing doses of (±)-SKF 82958 in naïve animals and when assessed in animals trained to self-administer cocaine [7, 16–19] failed to maintain SA responding above vehicle levels in rats previously trained to self-administer cocaine [20]. Nonetheless, these results suggest a substantial role of DA D1-like Rs in cocaine reinforcement.

### DA D2-like R direct full agonists

In contrast to the DA D1-like R full agonist (±)-SKF 82958, reinforcing effects of the DA D2-like R full agonists are not appreciable in naïve subjects. For example, none of doses of the DA D2-like R full agonist quinpirole maintained SA responding above vehicle levels in experimentally naïve rats [21] and in rats with a responding-reinforced history using food or (±)-ketamine [21]. Further, quinpirole was not reinforcing in rats trained to self-administer cocaine when the cocaine-paired stimulus was omitted from the contingency (i.e., SA responding resulted in quinpirole infusions without presentations of the previously cocaine-paired stimulus) [22]. In marked contrast, quinpirole [21–23] as well as other DA D2-like R full agonists [(±)-quinelorane [20,24], (±)-7-OH-DPAT [20,25], and pramipexole [25] all maintained high levels of responding when substituted for cocaine and delivered in conjunction with the cocaine-paired stimulus. The results from Drs. Woods and Collins are discussed more below (see Section *E*). In addition, a more recent study using the DA D_3_R full agonist PF-592,379 demonstrated a lack of reinforcing effects of the DA D_3_R full agonist when substituted for cocaine with response-dependent presentations of a cocaine-paired stimulus [9]. Thus, these results suggest the relatively limited role of DA D2-like Rs in mediating the reinforcing effects of cocaine, especially naïve subjects, which is quite distinct from the DA uptake inhibitors, such as cocaine [21,26] and methylphenidate [25], and the DA D1-like R full agonist (±)-SKF 82958 [16,17].

## Effects of genetic manipulation of DA receptors

Studies using a genetic disruption of the DA receptor subtypes in mice also have revealed the distinct role of each DA receptor subtype in cocaine SA under an FR schedule of reinforcement. Genetic disruptions of each DA receptor subtype could cause a developmental impairment and/or a functionally compensatory mechanism other than their physiological functions. However, consistent with results of assessment of a capacity of direct DA receptor full agonists to induce or maintain SA responding mentioned above (see Section *B*), it appears that DA D1-like Rs are undoubtedly more critical than DA D2-like Rs in the reinforcing effects of cocaine and perhaps another DA uptake inhibitors.

### DA D_1_R KO mice

For example, at doses that reliably maintained SA responding in drug-naïve wild-type (WT) mice, cocaine was virtually ineffective at maintaining SA responding in DA D_1_R KO mice that had been trained to nose-poke for food [18]. However, as a positive control, the μ-opioid receptor (MOR) full agonist remifentanil was reinforcing in DA D_1_R KO mice [18]. On the other hand, there is no report assessing cocaine SA using DA D_5_R KO mice at this point. Thus the results suggest D_1_Rs play an essential role in mediating the reinforcing effects of cocaine.

### DA D2-like R KO mice

In marked contrast to DA D_1_Rs [18], it appears that DA D2-like Rs are not necessary for cocaine to function as a reinforcer. For example, cocaine maintained comparable levels of SA responding in both DA D_2_R WT and KO mice, with only a modest rightward/upward shift of the descending limb of the DEC for cocaine SA observed in the DA D_2_R KO mice [24]. Similar to mice lacking DA D_2_Rs [24], genetic deletion of the DA D_3_R did not alter the reinforcing effects of cocaine as assess by either FR or progressive ratio (PR) schedules of reinforcement [27]. In addition, there was no appreciable difference in acquisition rates of SA responding with a fixed dose of cocaine (1.0 mg/kg/inj) between DA D_4_R KO mice and their WT controls [28]. Nonetheless, those findings from studies using DA D2-like R KO mice suggest that none of the DA D2-like R subtypes are strictly necessary to induce cocaine reinforcement. The conclusion from studies using DA D2-like R KO mice is in stark contrast to those using DA D_1_R KO mice, and suggest that in that DA’s effects at DA D_1_Rs, but not DA D_2_Rs, are required for cocaine reinforcement.

## Effects of pretreatment with DA receptor direct ligands on cocaine SA

The reinforcing effects of cocaine under an FR schedule of reinforcement in rats have been demonstrated to be substantially affected by transient pharmacological manipulations using direct DA receptor ligands given as a pre-session treatment. The results further indicate quite distinct role of DA D1-like and D2-like receptors in cocaine reinforcement (see Table 2 for details). Regarding DA direct agonists, only studies using full agonists are discussed since partial agonists generally can function as an antagonist against pharmacological effects of full agonists.

### DA D1-like R direct full agonists

In marked contrast to the standard DA uptake inhibitors [6,10–14], pretreatment with DA D1-like R full agonists produce a dose-dependent downward shift of the DECs for cocaine SA [12,20], suggesting an insurmountable antagonism of cocaine reinforcement. Importantly, the apparent antagonism of cocaine SA was relatively selective because the doses of DA D1-like R agonists that decreased cocaine SA did not alter food-maintained behavior under an identical schedule of reinforcement [12,20]. However, the insurmountable antagonism of cocaine SA with the D1-like R full agonists is not so surprising since the MOR full agonists (−)-morphine and (±)-methadone both also produce downward shifts of the DEC for SA of the MOR full agonist (−)-heroin [11]. Further, pre-feeding time-dependently shifted down rather than to the left a curve of responding maintained by presentations of different numbers of food pellets [11]. Thus these downward shifts in responding maintained by cocaine, (−)-heroin, and food may indicate satiation-like effects of the DA D1-like R full agonists. On the other hand, previous studies demonstrated an appreciable mechanism resulting in flattened curves theoretically [29] and practically [30], if only the ascending limb of DECs is sensitive to a pretreatment. It would be very important to assess whether the DA D1-like R agonism-induced decreases in cocaine SA are truly mediated through the DA D1-like Rs.

### DA D2-like R direct full agonists

As with standard indirect DA receptor agonists [6,10–14] but in marked contrast to the DA D1-like R full agonists [12,20], pretreatment with DA D2-like R full agonists dose-dependently shift the DECs of cocaine SA to the left [12,20]. In fact, the potentiation of cocaine reinforcement by DA D2-like R full agonists is indistinguishable from that produced by standard DA uptake inhibitors [6,10–14]. These findings may suggest a fundamental role of D2-like Rs in the maintenance of the reinforcing effects of cocaine. However, as discussed below (See Section *E*), the effects of DA D2-like R full agonists may be to enhance the conditioned reinforcing effects of cocaine-pared stimuli rather than the primary reinforcing effects of cocaine [21–23].

### DA receptor antagonists

The DA D1-like R antagonist SCH-39166 and the DA D2-like R antagonist *S* (−)-eticlopride dose-dependently shift the DECs of cocaine SA to the right, suggesting a competitive and typical antagonism of the reinforcing effects of cocaine [12,24,26,31]. Further, a combination with intermediate doses of the DA D1-like R antagonist SCH-39166 and the preferential D_2_R antagonist L-741,626 was more effective than either compound alone to antagonize cocaine SA [26]. In addition, L-741,626 produced a dose-dependent rightward shift of the DEC of cocaine SA [26] while the DA D_3/4_R antagonist L-745,829 and the DA D_4_R antagonist L-745,870 were ineffective against cocaine SA [24]. Thus, these findings suggest that the reinforcing effects of cocaine are mediated by a combined effect of DA at DA D1-like as well as D_2_ receptors.

## Effects of pretreatment with direct DA D2-like R ligands on responding maintained by presentations of a stimulus paired with cocaine injections

As mentioned above in a section D2, the potentiation of cocaine SA with the DA D_2-like_R full agonists [12,20] was indistinguishable from the effects of pretreatments with typical DA uptake inhibitors [6,10–14]. These findings may suggest that D2-like Rs are capable of modulating (i.e., enhancing) the primary reinforcing effects of cocaine; however, the results of a series of studies by Drs. Woods and Collins suggest an alternative mechanism. First, as noted above (section B.2) the apparent reinforcing effects of the DA D2-like R full agonist quinpirole were highly dependent upon experimental history, with quinpirole maintaining high rates of responding in rats that were trained to self-administer cocaine, and rates of responding that were no different than vehicle in rats that were either experimentally naïve, or trained to respond for food or (±)-ketamine [21]. Although the importance of reinforcement history in determining the reinforcing effects of DA D2-like R agonists has not been systematically evaluated, a history of cocaine SA is sufficient to establish the reinforcing effects of a number of structurally diverse DA D2-like R agonists [e.g., quinpirole [21–23], (±)-quinelorane [20,24], (±)-7-OH-DPAT [20,25], and pramipexole (Collins et al., 2012b)]. In addition to an appropriate reinforcement history, the apparent reinforcing effects of DA D2-like R agonists (quinpirole and pramipexole) also appear to depend on the response-contingent presentation of the stimuli that were previously paired with the training drug (e.g., cocaine-paired stimuli) [15,21–23]. Not only do DA D2-like R agonists fail to maintain SA responding if they are delivered without the cocaine-paired stimuli [21,22], but removing the cocaine-paired stimuli from the contingency after SA responding for a DA D2-like R agonist has been established results in a rapid extinction of responding [15]. Similarly, pretreatment of rats with DA D2-like R agonists dose-dependently increases responding maintained by presentations of a cocaine-paired stimulus, an effect that is not observed when food-paired or neutral stimuli are used as the maintaining event [15,21–23]. Together, these findings suggest the capacity of the D2-like R full agonists to maintain SA responding when substituted for cocaine SA [15,21–23] and of pretreatments of DA D2-like R agonists to shift the cocaine SA DECs to the left [12,20] results from a DA D2-like R agonist-induced enhancement of the conditioned reinforcing effects of the cocaine-paired stimulus rather than a primary reinforcing effect of the DA D2-like R agonist or from a DA D2-like R agonist-induced enhancement of the primary reinforcing effects of cocaine [32]. In addition to providing a mechanism for the apparent reinforcing effects of the DA D2-like R full agonist, such as relationship (i.e., DA D2-like Rs enhancing conditioned reinforcing effects) also provides an appreciable explanation for why cocaine is fully capable of functioning as a reinforcer in DA D2-like R KO mice [33].

## Summary

The reinforcing effects of cocaine are initiated by its inhibition of DA uptake, resulting in an enhancement of dopaminergic transmission in the central nervous system [34,35]. The reinforcing effects of cocaine are mediated though both DA D1-like and D2-like receptors; however, each of these two families of receptors has substantially distinct roles in mediating cocaine reinforcement. The literature cited above indicates an essential role of DA D1-like Rs in the primary reinforcing effects of cocaine. In marked contrast, the primary reinforcing effects of cocaine can be produced without DA D2-like Rs. However, Drs. Woods and Collins demonstrated the appreciable capacity of the DA D2-like R full agonists to dose-dependently enhance the conditioned reinforcing effects of cocaine-paired stimulus, but not cocaine *per se*. These findings are useful in understanding how DA D2-like R antagonists can antagonize several effects of various compounds across pharmacological classes including methamphetamine, opioids, ethanol, nicotine, and non-competitive NMDA glutamate receptor antagonist. Given a lack of effects of DA receptor antagonists on SA of (−)-heroin and (±)-ketamine (Hiranita et al., 2013b), DA D2-like Rs might likely contribute to maintenance of D2-like R-mediated conditioning effects of (−)-heroin and (±)-ketamine, but not to their primary reinforcing effects since (−)-heroin and (±)-ketamine can increase DA levels in brains. Further these findings could have a substantial impact on a psychiatric research field since DA is involved in a wide range of various psychiatric diseases and symptoms.

## Figures and Tables

**Table 1:** Self-administration (SA) of DA receptor agonists in rats and mice. SA was defined as operant responding maintained above levels of vehicle injections. Unless described, drug injections were response-dependently accompanied with a stimulus change DEC, dose-effect curve.

Pharmacological Class	Self-administered Drug	Reinforcement history	Primary finding (reference)
DA releaser	*d*-Methamphetamine	None	Dose-dependently maintained SA responding above vehicle levels in rats [26]
		(−)-Heroin	Dose-dependently maintained SA responding above vehicle levels in rats [11]
		(±)-Ketamine	Dose-dependently maintained SA responding above vehicle levels in rats [11]
	*d*-Amphetamine	Cocaine	Dose-dependently maintained SA responding above vehicle levels in mice [8] Dose-dependently maintained SA responding above vehicle levels and virtually without any shift in a cocaine SA DEC in DAT KI mice [8]
		*d*-Methamphetamine	Dose-dependently maintained SA responding above vehicle levels in rats [11,26]
		(−)-Heroin	Dose-dependently maintained SA responding above vehicle levels in rats [11]
		(±)-Ketamine	Dose-dependently maintained SA responding above vehicle levels in rats [11]
DA uptake inhibitor	Cocaine	None	Dose-dependently maintained SA responding above vehicle levels in rats [11,21] **Failed to maintained SA responding above vehicle levels in DA D_1_R KO mice** [18]
		Food	Maintained SA responding above vehicle levels in rats [21] **None of the doses maintained SA responding above vehicle levels in DAT KO mice** [7] **None of the doses maintained SA responding above vehicle levels in DAT KI mice** [8] **None of the doses maintained SA responding above vehicle levels in DA D_1_R KO mice** [18] **None of the doses maintained SA responding above vehicle levels in DA D_4_R KO mice** [28]
		*d*-Methamphetamine	Dose-dependently maintained SA responding above vehicle levels in rats [11]
		(−)-Heroin	Dose-dependently maintained SA responding above vehicle levels in rats [11]
		(±)-Ketamine	Dose-dependently maintained SA responding above vehicle levels in rats [11]
	Methylphenidate	None	Dose-dependently maintained SA responding above vehicle levels in rats [25]
		Cocaine	Dose-dependently maintained SA responding above vehicle levels in rats [6,10]
	Nomifensine	Cocaine	Dose-dependently maintained SA responding above vehicle levels in rats [10]
	RTI-366	Cocaine	Dose-dependently maintained SA responding above vehicle levels in rats [10]
	WIN 35,428	Cocaine	Dose-dependently maintained SA responding above vehicle levels in rats [10]
		*d*-Methamphetamine	Dose-dependently maintained SA responding above vehicle levels in rats [11]
		(−)-Heroin	Dose-dependently maintained SA responding above vehicle levels in rats [11]
		(±)-Ketamine	Dose-dependently maintained SA responding above vehicle levels in rats [11]
DA D1-like R full agonist	(±)-SKF 82958	Food	Dose-dependently maintained SA responding above vehicle levels in rats [16] Dose-dependently maintained SA responding above vehicle levels in DAT KO mice [7] **None of the doses maintained SA responding above vehicle levels in DA D_1_R KO mice** [18]
		Cocaine	**None of doses tested maintained SA responding above vehicle levels in rats** [20] Dose-dependently maintained SA responding above vehicle levels in mice[18] Maintained SA responding above vehicle levels in DAT KI mice [8]
DA D2-like R full agonist	(±)-7-OH-DPAT	Cocaine	Dose-dependently maintained SA responding above vehicle levels in rats [12,20]
	Pramipexole	Cocaine	Dose-dependently maintained SA responding above vehicle levels in rats [15]
	(±)-Quinelorane	Cocaine	Dose-dependently maintained SA responding above vehicle levels in rats and mice [18,20,24]
	Quinpirole	None	**Failed to maintained SA responding above vehicle levels even in rats when response-** **paired stimulus was presented response-dependently** [21]
		Food	**Failed to maintained SA responding above vehicle levels in rats even when food-** **paired stimulus was presented response-dependently** [21]
		Cocaine	Maintained SA responding above vehicle levels in rats [22,23] **Failed to maintained SA responding above vehicle levels in rats when cocainepaired stimuli were omitted** [22]
		Remifentanil	Dose-dependently maintained SA responding above vehicle levels in rats [21]
		(±)-Ketamine	**Failed to maintained SA responding above vehicle levels in rats even when** **(±)-ketamine -paired stimulus was presented response-dependently** [21]
DA D_3_R full agonist	PF-592,379	Cocaine	**None of doses tested maintained SA responding above vehicle levels in rats** [9]

**Table 2:** Effects of pretreatment with DA receptor agonists in rats and mice. Unless described, drug injections were response-dependently accompanied with a stimulus change.

Pharmacological Class	Pretreated drug	Primary finding (reference)
DA releaser	*d*-Amphetamine	Dose-dependent leftward shifts in a cocaine SA DEC in rats [12]
		Dose-dependent leftward shifts in a *d*-methamphetamine SA DEC in rats [14]
DA uptake inhibitor	Cocaine	Leftward shifts in a cocaine SA DEC [12]
	GBR 12909	Dose-dependent leftward shifts in a cocaine SA DEC in rats [12]
	Methylphenidate	Dose-dependent leftward shifts in a cocaine SA DEC in rats [6,10,13]
	Nomifensine	Dose-dependent leftward shifts in a cocaine SA DEC in rats [10]
	RTI-366	Dose-dependent leftward shifts in a cocaine SA DEC in rats [14]
	WIN 35,428	Dose-dependent leftward shifts in a cocaine SA DEC in rats [10]
		Dose-dependent leftward shifts in a *d*-methamphetamine SA DEC in rats [11]
DA D1-like R full agonist	(±)-SKF 82958	Dose-dependent downward shifts in a cocaine SA DEC in rats [12,20]
	*R*(+)-6-Br-APB	Dose-dependent downward shifts in a cocaine SA DEC in rats [12]
DA D1-like R antagonist	SCH 23390	Right-shifted a cocaine SA DEC in mice [18]
	SCH 39166	Dose-dependent rightward shifts in a cocaine SA DEC in rats [12,26]
DA D2-like R full agonist	(±)-7-OH-DPAT	Dose-dependent leftward shifts in a cocaine SA DEC in rats [12,20]
	(±)-Quinelorane	Dose-dependent leftward shifts in a cocaine SA DEC in rats [12,20]
	quinpirole	Increased SA responding maintained by cocaine with response-dependent presentations of cocaine-paired stimulus in rats [22]
		**Without effects on SA responding maintained by cocaine without presentations of cocaine-paired stimuli in rats** [22]
DA D2-like R antagonist	*S*(−)-Eticlopride	Dose-dependent rightward shifts in a cocaine SA DEC in rats [12,24,31]
Preferential D_2_R antagonist	L-741,626	Dose-dependent rightward shifts in a cocaine SA DEC in rats [26]
DA D_3/4_R antagonist	L-745,829	**Decreased only an ascending limb of a cocaine SA DEC in rats** [24]
DA D_4_R antagonist	L-745,870	**Virtually without a shift in a cocaine SA DEC in rats** [24]

## Non-standard Abbreviations

DA: Dopamine
DAT: Dopamine Transporter
D_1_R: D_1_ Receptor
D_2_R: D_2_ Receptor
D_3_R: D_3_ Receptor
D_4_R: D_4_ Receptor
D_5_R: D_5_ Receptor
D_1-like_R: D_1_-Like Receptor
D_2-like_R: D_2_-Like Receptor
DEC: Dose-effect Curve
FR: Fixed Ratio
KI: Knockin
KO: Knockout
MOR: μ-Opioid Receptor
PR: Progressive Ratio
SA: Self-Administration
WT: Wild-Type
